# Self-Calibrated Humidity Sensor in CMOS without Post-Processing

**DOI:** 10.3390/s120100226

**Published:** 2011-12-27

**Authors:** Oleg Nizhnik, Kohei Higuchi, Kazusuke Maenaka

**Affiliations:** 1 Maenaka Human-sensing Fusion Project, 8111, Shosha 2167, Himeji-shi, Hyogo-ken, Japan; E-Mail: k-higuchi@eratokm.jp; 2 Hyogo Prefectural University, 8111, Shosha 2167, Himeji-shi, Hyogo-ken, Japan; E-Mail: maenaka@eng.u-hyogo.ac.jp

**Keywords:** capacitive sensors, microsensors, humidity measurement, CMOS integrated circuits

## Abstract

A 1.1 μW power dissipation, voltage-output humidity sensor with 10% relative humidity accuracy was developed in the LFoundry 0.15 μm CMOS technology without post-processing. The sensor consists of a woven lateral array of electrodes implemented in CMOS top metal, a humidity-sensitive layer of Intervia Photodielectric 8023D-10, a CMOS capacitance to voltage converter, and the self-calibration circuitry.

## 1. Introduction

The history of humidity sensor integration with CMOS is at least 23 years old [1]. After nearly a quarter of a century of development, three main sensing designs have emerged. Besides the water-absorbing film-based solution in [1], a design based on the thermal conductivity difference between air and water vapor was also proposed in [2]. For very precise systems, dew point can be also measured with the hybrid CMOS/MEMS chip [3]. For mass-produced humidity sensors, like those found in sensor networks [4], low power dissipation and low price is obligatory. To reduce price, the amount of CMOS post-processing and packaging should be kept to a minimum. Existing proposals for CMOS-based sensors require a porous metal on the top layer [1], or complex multilayer geometries over the CMOS wafer [5,6]. In [7], by virtue of using CMOS with a thick top metal option, only one additional (sensing polymer) layer is deposited. However, because the sensing element is patterned, a patterning mask aligned with metal layers is still needed to define the sensing layer. In [8], the authors have investigated the possibility of using the standard passivation layer of the CMOS chip as the humidity sensing element with electrode shapes similar to [5,6], but using CMOS thick metal in a fashion similar to [7]. The implementation resulted in a functional humidity sensor with voltage output described in [8]. Unfortunately, large (50 RH %) random offset of the output, low gain (0.3 mV/RH %) and freezing stress-induced delamination of the sensing layer have made the humidity sensor design in [8] less than useful. In the current work, a capacitance subtraction circuit (to improve the gain of the C/V converter), a voltage offset sensor (self-calibration system) and a stress reducing guard ring were added to implement a useful humidity sensor.

## 2. Circuits and Layout Description

**Figure 1.**
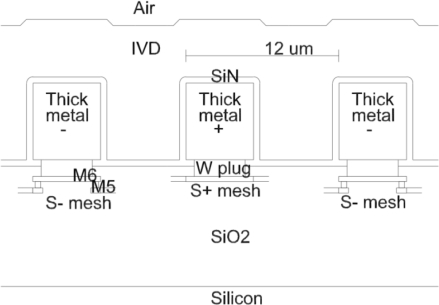
Side-view cross-section of the humidity sensor.

Humidity sensors using hygroscopic polymer films are the most suitable for integration in CMOS. A hard-cured polymer film absorbs moisture, increasing its effective dielectric constant with humidity in predictable and reversible process. But this approach has problems to solve. Firstly, hygroscopic films produce humidity-dependent capacitance, so a capacitance-to-voltage on-chip converter is necessary for simplified processing of the sensor output. Secondly, the implementation of the sensing element is not straightforward. To obtain a large relative capacitance change, electrical flux must be confined to the hygroscopic film. The simplest way to reach this objective is if the electrodes enclose the hygroscopic film from all sides. But in order for moist air to access the sensing film, there must be a large area of the polymer exposed to the air. In the implemented humidity sensor, electrical flux reaching the CMOS substrate was confined by a woven electrode mesh below the main sensing electrodes (see Figure 1). The woven mesh creates a 2-dimenational multi-pole field distribution, falling exponentially below mesh. With the implemented mesh geometry, a 60% electrode-to-substrate capacitance reduction is expected. This is more effective compared to the classical comb structure, producing a 1-dimensional multi-pole field, which is expected to close only 46% of the electrical flux lines above the substrate.

**Figure 2.**
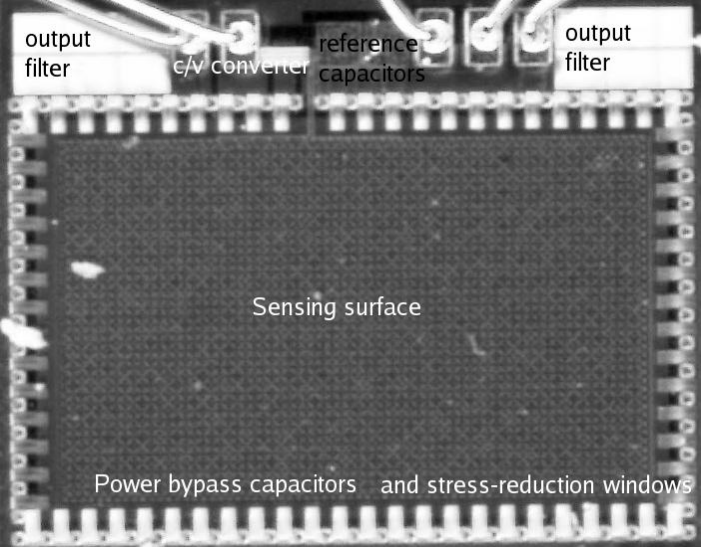
Microphotograph of the humidity sensor.

Furthermore, the previously proposed comb-type electrodes have a metal density of 50% if sensitivity is maximized [6]. For the LFoundry 0.15 μm CMOS, the specified maximal thick metal density is 10%. Therefore, in the proposed design, an electrode pattern with CMOS thick metal density of 20% and sensitivity equal to the classical comb pattern was developed (see Figure 2). Positive and negative cube-like electrodes were placed in a checkerboard pattern. The humidity-sensing polymer was an 11 μm thick coating of “Intervia Dielectric 8023D-10”, included in the LFoundry CMOS process as part of the pads mask. This material has a specified moisture absorption of 0.5% and a relative dielectric permittivity equal to 3.2. A sensor with comb-shaped electrodes was also manufactured. Although free of defects, large capacitance to the substrate has resulted in saturation of the C/V converter.

The design in [8] was compromised by delamination of the sensing film at low temperatures and high humidity, likely because of the water freezing in the gap between IVD film and electrodes. The ring of small square openings in IVD around the humidity sensing area reduces the stress of dicing transferred to IVD in sensing area, therefore preventing the initial gap formation. These windows also act as a crack stopper, preventing delamination occurring on other parts of the chip from reaching the humidity sensor. Elimination of that initial gap has resulted in reduction of minimal tolerated temperature (−20 °C instead of 5 °C in [8]) and increase of maximal measurable humidity (100% instead of 95% in [8]). The photograph of the newly fabricated humidity sensor is shown in Figure 2.

**Figure 3.**
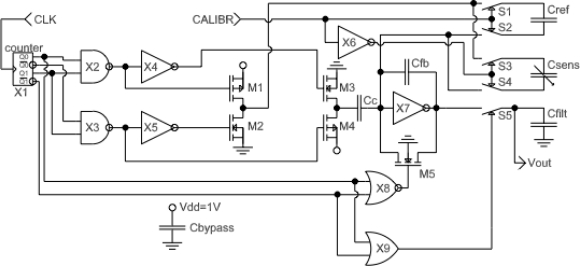
Schematic of the CMOS humidity sensor.

**Figure 4.**
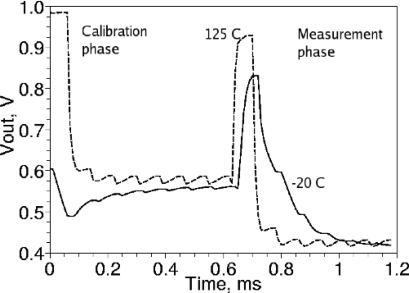
The simulated performance of the C-V converter at different temperatures.

A C/V (capacitance-to-voltage) converter derived from [9] was developed. Compared to the design in [9], the circuit was adapted for differential operation, and a sample-and-hold circuit was implemented at the output instead of a multiplexer. The design schematic without ESD diodes and pad drivers is shown in Figure 3. Compared to the design in [8], an internal synchronous 2-bit counter was added to simplify the control waveforms (now only one input clock is obligatory) and to reduce crowbar current, resulting in a power dissipation reduction of 30%. Although a simple D-flip-flop in place of the synchronous counter would reduce transistor count by 26, Monte-Carlo simulation has demonstrated that with a single D-flip-flop, output glitches may cause an unpredictable offset voltage at Vout, up to the saturation point of the C/V converter. The new circuits are the self-calibration system (components in Figure 3 are S1, S2, X6) and constant capacitance cancellation system (components in Figure 3 are Cc, M3, M4, X2-X5). Capacitance cancellation works by subtracting charge from the input node of the C/V converter (components on Figure 3 are Cfb, X7, M1, M2, M5) through the fixed MIM capacitor Cc. With constant capacitance roughly cancelled, the size of the feedback capacitor Cfb can be reduced, improving the gain of the C/V converter without risking C/V converter saturation. The self-calibration system connects the input of the C/V converter to the built-in MIM capacitor reference Cref instead of the humidity sensing capacitor if CALIBR input is set to high. The primary role of the self-calibration is the measurement of the random mismatch of the transistors in C/V converter. Mismatch-dependent voltage is presented on the output Vout of the C/V converter. An additional function of the self-calibration circuit is the tracking of the temperature-voltage coefficient (TCV) of the C/V converter which allows the cancellation of the TCV, as illustrated in Figure 4.

Figure 4 shows the simulated voltage at the output of the C-V converter at −20 °C and +125 °C. The temperature-dependent offset voltage affect the steady-state output nearly equally in the calibration and measurement modes, allowing for the reduction of the temperature-voltage coefficient if calibration voltage is taken into account.

## 3. Experimental Results

Experimental results for manufactured humidity sensors are summarized in Table 1.

**Figure 5.**
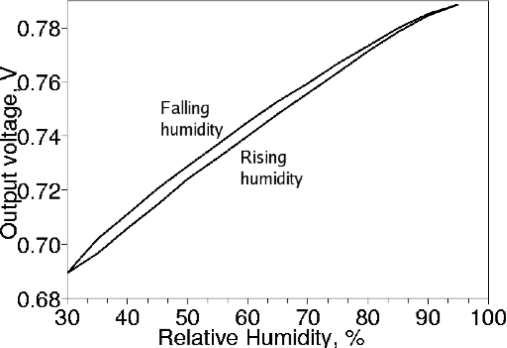
Hysteresis of humidity sensor at 25 °C.

**Figure 6.**
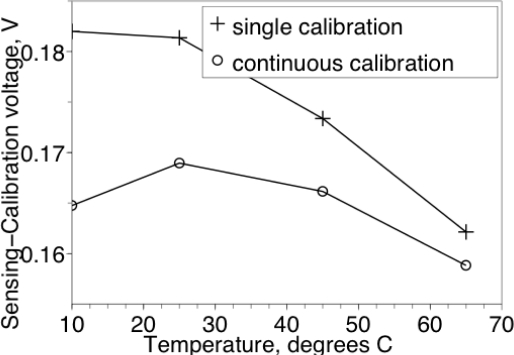
Temperature dependence of the sensor output at RH = 50%. Single-calibration curve shifted 10 mV for clarity.

Figure 5 shows the typical absorption-desorption characteristic of a single humidity sensor. Although both the output voltage at 50% RH and the calibration voltage were designed to be Vdd/2 = 0.5 V, inaccurate model extraction from the measurements of the sensor [8] resulted in 0.68–0.72 V output voltage and 0.44–0.48 V calibration voltage. Although the calibration voltage has a 40 mV spread between chips in the same batch, the difference between calibration voltage and the sensing voltage at 0% RH was between 159 mV and 163 mV for all 5 chips in the batch. Furthermore, the temperature coefficient of the difference between calibration voltage and sensing voltage was reduced, as illustrated in Figure 6. Therefore, the calibration voltage can be used to cancel out the random mismatch of the C/V converter with a reduction coefficient of 10:1, and also allows the reduction of temperature dependence of the readout by approximately two-fold. Such reduction of the offset errors allows 10% accuracy of the relative humidity measurements without external calibration standards, greatly simplifying the manufacture, installation and the usage of the humidity sensor.

The accuracy mentioned in the Table 2 is defined as the nonlinearity plus half the hysteresis. The 70-second response time of designed sensor is slow compared to other designs, but still more than enough for environmental monitoring. All measurement was done using temperature-humidity chamber SH-24. After 90 cycles (1 month) of the humidity (30% to 95%) and 30 cycles of the temperature (−20 to 120 °C) the drift was found to be 9% RH. For the tested sample, the main effect of cycling is the increased C/V converter gain (1.58 to 1.78 mV/RH %) and decreased voltage at 0% RH (641 to 628 mV). This effect is most likely caused by chemical bonding of water to the sensing polymer film near the maximal temperature, causing irreversible swelling of the film. Versions with the smaller capacitor Cc to get larger gain was fabricated, but was found saturating due the insufficient accuracy of the sensing element model. Further refinement of the sensing element model may result in increase of the gain up to four-fold compared to the current model.

## 4. Conclusions

An ultra-low power, low-cost, self-calibrated humidity sensor in CMOS without post-processing was designed and evaluated. Simple control and readout of the proposed sensor allows its usage in low-end sensor nodes and embedded systems. The sensor can work with an RC-oscillator and a low-cost MCU to comprise a complete sensing system. The performance of the developed humidity sensor is summarized in Table 1 and compared with other humidity sensors for embedded applications in Table 2.

## Figures and Tables

**Table 1.** Performance summary 2nd batch of five humidity sensors compared with earlier design.

**Parameter**	**Min.**	**Avg.**	**Max.**	**Min [8]**	**Avg [8]**	**Max [8]**
Humidity range, RH %	10 *	-	100	10 *	-	95
*Operating temperature range, °C*	−20 #	-	120 #	5	-	120 #
*Hysteresis, RH %*	2.7	3.1	4.0	4.4	5.5	6.6
*TCV, RH %/C, w/o calibration*	−0.25	−0.19	0.04	−0.61	0.07	0.19
*TCV, RH %/C, after subtracting cal. voltage*	−0.06	−0.04	0.16	-	-	-
*Nonlinearity (best fit straight line), RH %*	1.5	2.2	3.6	1.1	1.3	1.7
*Drift (after 30 1-day cycles −20 to 120 °C), RH %*	-	-	9	-	-	-
Response time, 1/e at 95% to 45% RH change, s	-	70	-	-	70	-
Voltage at 0% RH minus calibration voltage, mV	148	160	163	146	160	168
*Gain, mV/RH %*	1.74	1.77	1.8	0.34	0.37	0.39
*Output impedance, MOhm*	-	1	-	-	1	-
Power dissipation at 1 V power supply, μW	0.9	1.1	1.5	1.3	1.6	1.9
Power supply voltage range, V	0.8	1	1.8	0.8	1	1.8
Chip area with pads, mm^2^ (0.1 × 0.1 mm pads)		0.70		-	0.52	-

* Limited by dryer capability of humidity chamber;

# Due to reference humidity meter limitations, performance measurements were done only in the range 5–100 °C. Drift test was only in range −20–120 °C.

**Table 2.** Comparison of the humidity sensors.

**Device name**	**Power**	**Accuracy**	**Price**
This work	1.1 μW	10% #	2.3 USD *
[8]	1.6 μW	5%	1.7 USD *
[4] (SHT11)	28 μW	5%	36 USD
[10] HIH5030	540 μW	5%	6 USD
[11] CHS-UGS	3 mW	5%	17 USD

* Estimated for mass production;

# 6% if external 2-points calibration is used.
